# Double Jeopardy: Spontaneous Coronary Artery Dissection and Stress-Induced Cardiomyopathy

**DOI:** 10.7759/cureus.117254

**Published:** 2026-09-30

**Authors:** Gabriel Gueyikian, Amir Darki

**Affiliations:** 1 Biology, Loyola University Chicago, Chicago, USA; 2 Interventional Cardiology, Loyola University Medical Center, Maywood, USA

**Keywords:** acute coronary syndrome, dissection, spontaneous coronary artery dissection, stress induced cardiomyopathy, takotsubo syndrome

## Abstract

Spontaneous coronary artery dissection (SCAD) and stress-induced cardiomyopathy (SIC), or Takotsubo syndrome, are distinct nonatherosclerotic causes of acute coronary syndrome that predominantly affect women and share physical and emotional triggers. Their simultaneous occurrence is uncommon and presents diagnostic and therapeutic challenges. We report a 74-year-old woman with hypertension and a prior cerebrovascular accident who presented with severe, persistent chest pain and a high-sensitivity troponin level of 9,143 ng/L. She was diagnosed with non-ST-segment elevation myocardial infarction and treated with intravenous heparin and nitroglycerin. Because of persistent symptoms and high-risk features, urgent coronary angiography was performed and demonstrated SCAD involving the apical left anterior descending coronary artery. Echocardiogram demonstrated apical ballooning with preserved basal contractility, raising concern for concomitant SIC. Myocardial infarction due to SCAD remained in the differential diagnosis. The patient was initially hemodynamically stable following catheterization with plans to obtain a cardiac MRI for further evaluation; however, on hospital day 2, she developed complete heart block that progressed to asystolic cardiac arrest, and she died despite prolonged resuscitative efforts. This case highlights the potential coexistence of SCAD and SIC, and emphasizes the importance of carefully evaluating patients with apparent Takotsubo cardiomyopathy for concomitant coronary pathology. It also illustrates the potential electrical and clinical complications associated with these overlapping conditions and supports a possible shared catecholamine-mediated or bidirectional pathophysiologic relationship.

## Introduction

Spontaneous coronary artery dissection (SCAD) is an increasingly recognized cause of acute coronary syndrome (ACS), characterized by a spontaneous tear or bleeding within the coronary arterial wall that generates an intramural hematoma and restricts blood flow [1]. Stress-induced cardiomyopathy (SIC), also known as Takotsubo syndrome (TTS), is defined by transient left ventricular systolic dysfunction, most often apical ballooning, in the absence of obstructive coronary artery disease. Both conditions share overlapping clinical features, including chest pain, electrocardiographic changes, elevated cardiac biomarkers, and a predilection for women, frequently following physical or emotional stressors [2]. We report a case of a 74-year-old woman who presented with SCAD and possible concomitant stress-induced cardiomyopathy, highlighting their clinical overlap and potential mechanistic association.

## Case presentation

A 74-year-old woman with a past medical history of cerebrovascular accident and hypertension presented to the emergency department with 3 hours of severe, persistent chest pain. On arrival, her vital signs included a blood pressure of 169/82 mmHg, heart rate of 93 beats per minute, and oxygen saturation of 95% on room air. Laboratory testing revealed a markedly elevated high-sensitivity troponin concentration of 9,143 ng/L (reference range, 0-14 ng/L). Electrocardiography demonstrated sinus bradycardia with anterolateral T wave inversions (Figure 1).

**Figure 1:**
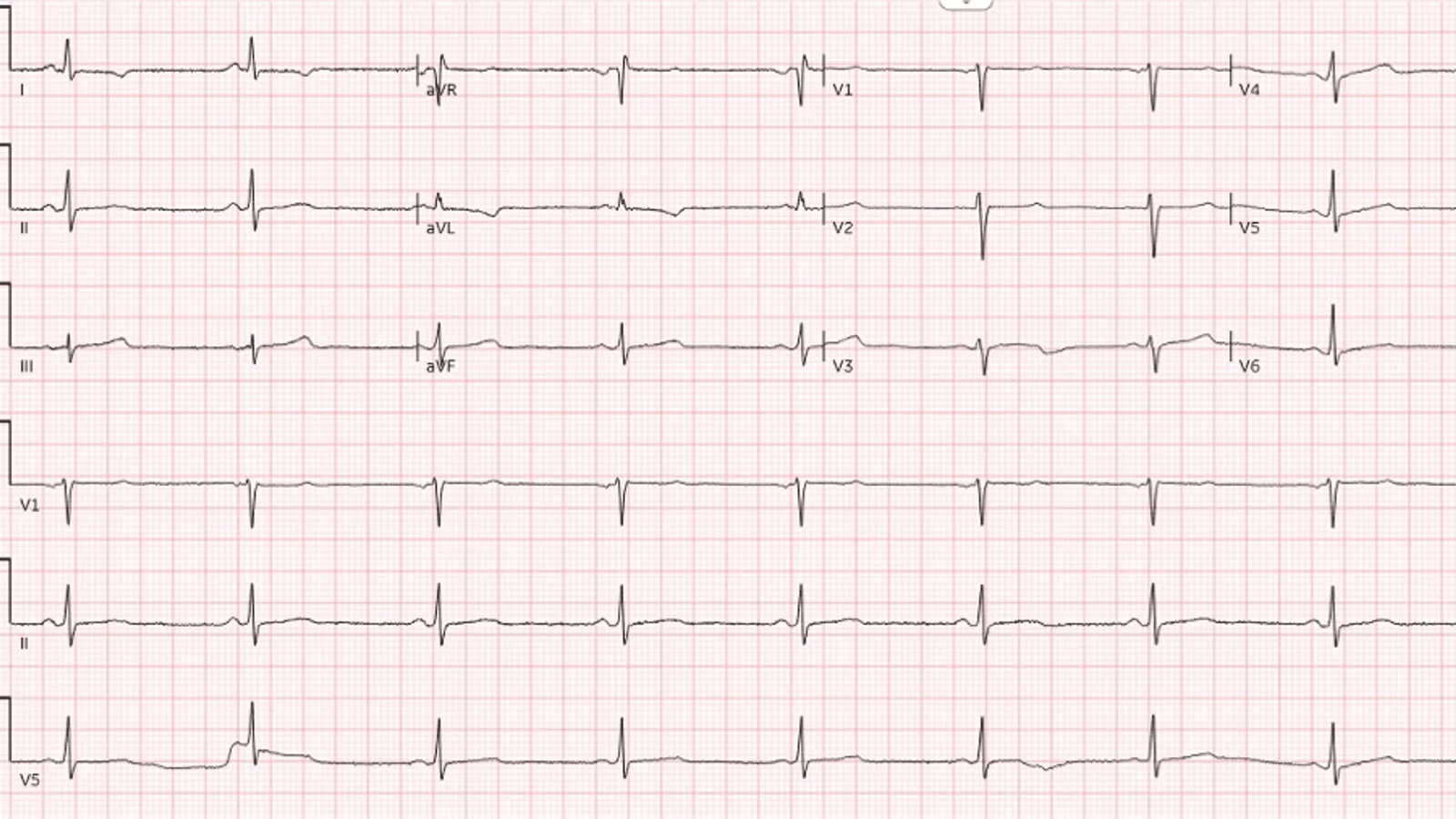
Electrocardiogram demonstrating sinus bradycardia with anterolateral T wave flattening/inversions

Given the clinical presentation, she was diagnosed with a non-ST-segment elevation myocardial infarction (NSTEMI). Intravenous unfractionated heparin and nitroglycerin infusions were initiated. Despite medical therapy, the patient continued to experience chest pain. In the setting of ongoing ischemia, significantly elevated cardiac biomarkers, and high-risk features, an early invasive strategy with coronary angiography was recommended.

Invasive coronary angiography demonstrated spontaneous coronary artery dissection (SCAD) involving the apical segment of the left anterior descending (LAD) artery (Figure 2).

**Figure 2:**
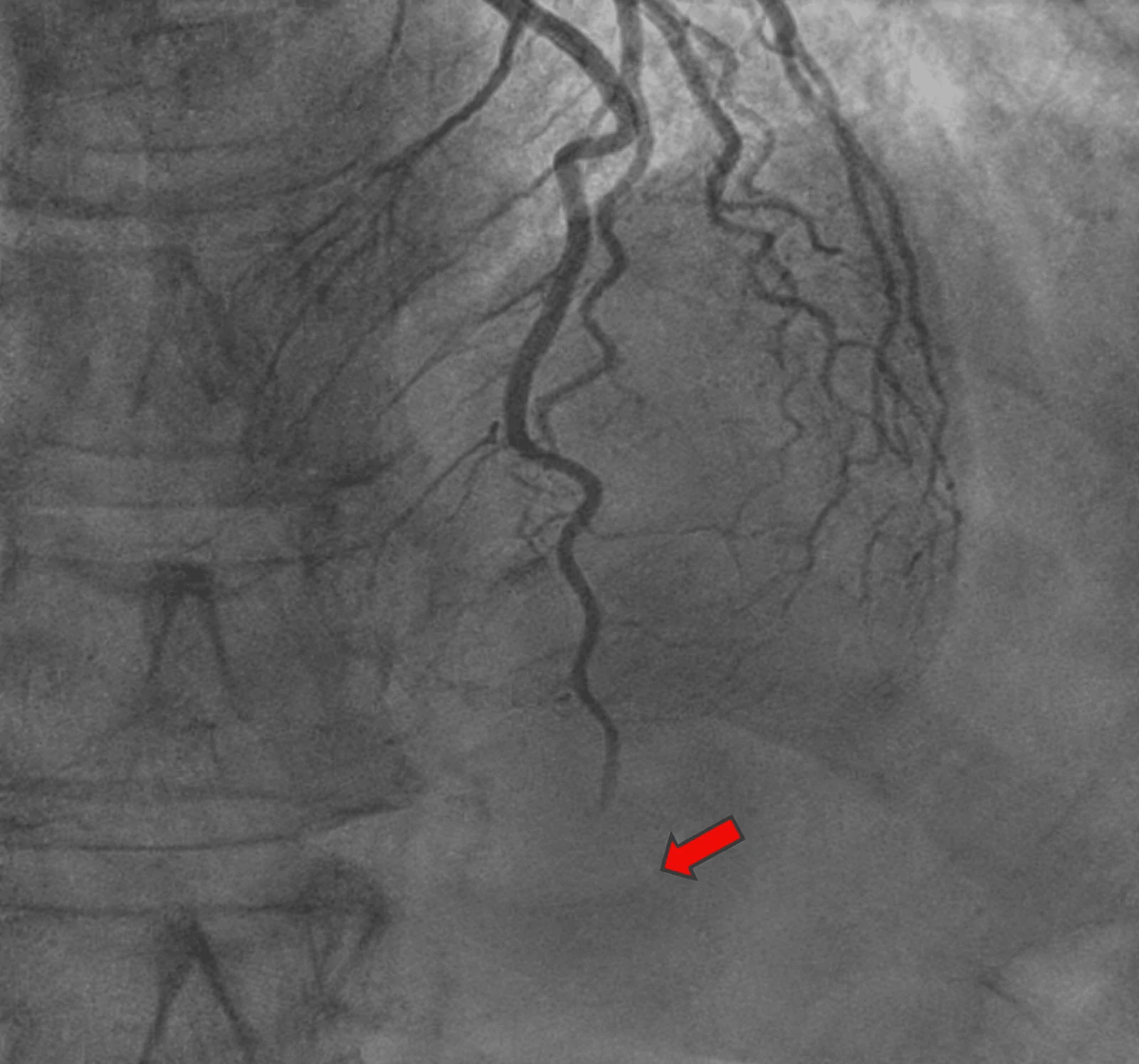
Distal LAD SCAD The arrow indicates spontaneous coronary artery dissection of the distal left anterior descending (LAD) coronary artery, resulting in complete vessel occlusion with absent distal flow (TIMI 0), consistent with type 4 spontaneous coronary artery dissection (SCAD).

Echocardiogram revealed findings suggestive of stress-induced (Takotsubo) cardiomyopathy, characterized by apical ballooning with relative preservation of basal contractility (Figure 3).

**Figure 3:**
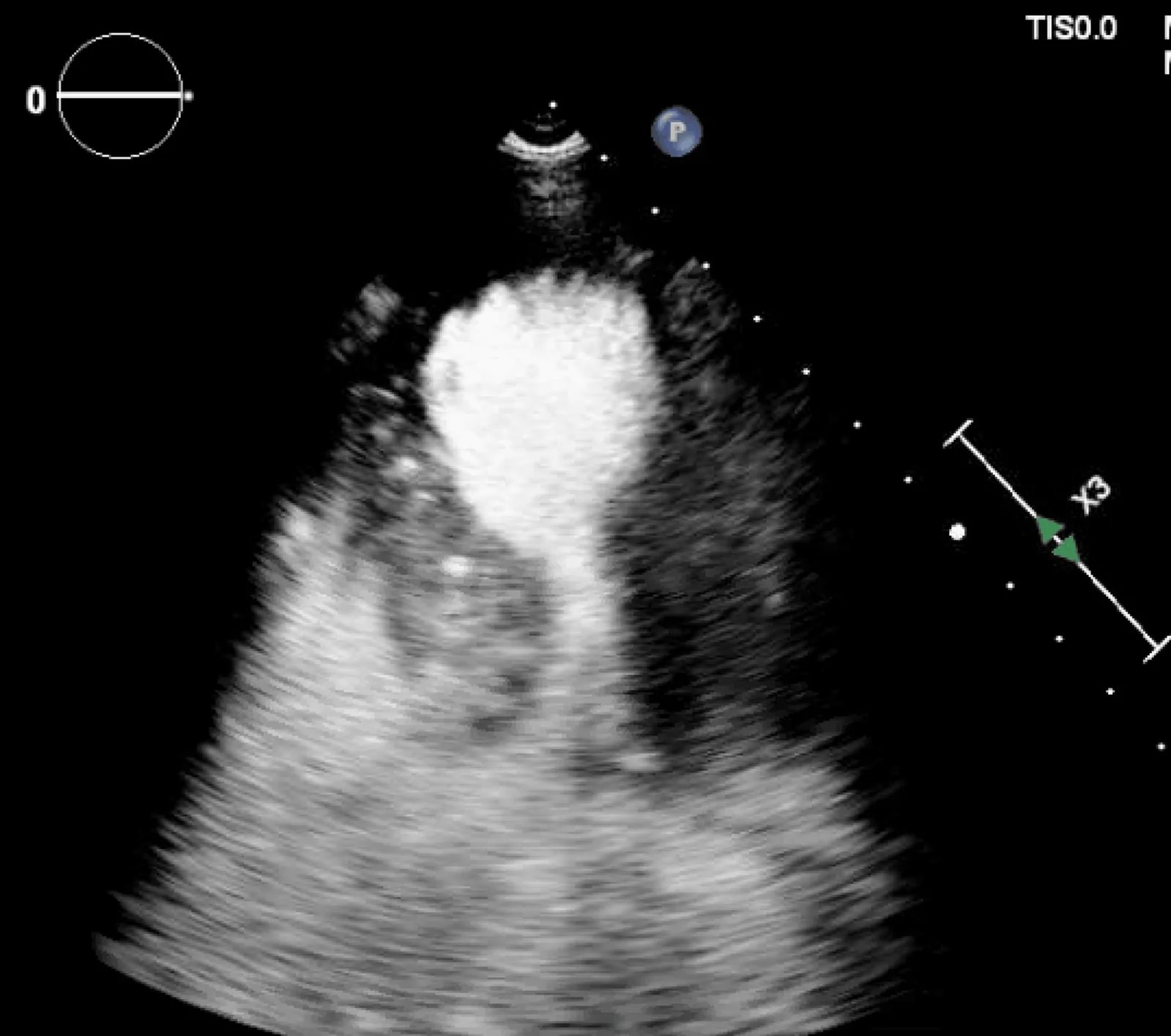
Stress-induced cardiomyopathy Contrast-enhanced transthoracic echocardiography demonstrating apical ballooning with preserved basal contractility, suggestive of stress-induced (Takotsubo) cardiomyopathy.

The patient was admitted to the intensive care unit for close monitoring. Initially, the patient was hemodynamically stable, and laboratory results were unchanged, with plans to obtain a cardiac MRI for further evaluation. Unfortunately, on hospital day 2, the patient developed complete heart block that progressed to asystole. Advanced cardiovascular life support was initiated immediately; however, the patient remained in asystole throughout the 20-minute resuscitation attempt.

## Discussion

SCAD and SIC are two distinct, increasingly recognized nonatherosclerotic causes of acute coronary syndrome. Both predominantly affect women and are associated with physical and emotional stressors. Although their clinical overlap is well-documented, their simultaneous occurrence is uncommon. We present a rare case of concurrent SCAD and possible SIC in a 74-year-old woman.

SCAD and SIC share several clinical features that can complicate their differentiation. Both conditions may present with chest pain, electrocardiographic abnormalities, and elevated cardiac biomarkers, including troponin [1]. The left anterior descending coronary artery is most commonly affected in SCAD, potentially producing left ventricular wall-motion abnormalities that are indistinguishable from those characteristic of SIC [2]. These similarities may contribute to misdiagnosis. Among patients with a provisional diagnosis of Takotsubo syndrome (TTS), 2.5% had definitive angiographic evidence of SCAD upon expert review, while an additional 9% required further imaging to confirm or exclude SCAD [2].

Emotional and physical stressors have consistently been identified as potential precipitating factors in SCAD, occurring in approximately 57%-66% of cases reported in the literature. Among patients with concurrent SCAD and SIC, a precipitating stressor was identified in approximately 57% of cases [3].

SCAD is a mechanistically distinct cause of myocardial infarction resulting from spontaneous, intimal disruption of the coronary wall rather than atherosclerotic plaque rupture [1]. The traditional cardiovascular risk factors that guide the recognition of conventional acute coronary syndrome are often absent [1]. Both SCAD and SIC predominantly affect women, although they differ in their typical age distributions. SCAD is most commonly reported in women younger than 50 years, whereas SIC predominantly affects postmenopausal women [2]. The present case is therefore unique, as it describes a patient who was 74 years old and had a history of hypertension and prior cerebrovascular accident, a demographic and clinical profile not typically associated with SCAD. This case highlights the importance of maintaining a broad differential diagnosis for acute coronary syndrome, including SCAD, even among older patients with conventional cardiovascular risk factors [4].

The mechanistic relationship between SCAD and SIC remains incompletely understood, with current evidence supporting the plausibility of bidirectional causation [3,4]. Two principal hypotheses have been proposed. The first suggests that SIC may precipitate SCAD through mechanical disruption of the coronary arterial wall. The characteristic wall-motion pattern of SIC, in which the basal myocardium contracts forcefully while the midventricular and apical segments remain akinetic, creates substantial mechanical stress at the interface between these regions. In susceptible individuals, the resulting force at this “hinge point” may disrupt the overlying coronary arterial wall, leading to dissection or intramural hematoma formation [3,4]. This proposed mechanism has been termed acute mechano-cardiac coronary artery disruption (AMCAD).

Conversely, SCAD itself may trigger SIC. The symptoms of SCAD: chest pain, shortness of breath, and hemodynamic instability represent a significant physical and emotional stressor capable of provoking the catecholamine surge underlying SIC. This is similar to the phenomenon of TTS occurring in the setting of acute myocardial infarction, where the ischemic event itself initiates SIC [4]. The directional relationship can be confirmed by cardiac MRI showing that transmural infarction in the territory of the dissected vessel alongside the broader apical wall motion pattern of SIC demonstrated the SCAD-caused ST-elevation MI that subsequently triggers TTS [4].

Underlining both hypotheses is a shared biological pathway. Excess catecholamines and sympathetic nervous system activation are implicated in both conditions. These processes may cause direct heart muscle injury and abnormal narrowing of the coronary arteries in SIC, and increased stress on the coronary artery wall in SCAD [4]. Both conditions are associated with anxiety, depression, migraines, hormonal changes, and stressful life events, suggesting that certain individuals may be inherently vulnerable to either or both conditions [1]. However, association does not equal causation, and in many cases of concurrent SCAD and SIC, a shared trigger, rather than one condition causing the other, may account for their simultaneous existence [4].

The initial evaluation for SCAD and SIC follows the standard approach for suspected acute coronary syndrome, beginning with electrocardiography (ECG). However, ECG findings in SCAD are nonspecific and may overlap with those of atherosclerotic myocardial infarction and SIC, limiting their diagnostic utility in isolation [1,2].

Transthoracic echocardiography plays an important diagnostic role, as demonstrated in this case, in which it revealed apical ballooning with preserved basal contractility-findings characteristic of SIC. This finding, however, warrants careful interpretation: among patients with a provisional diagnosis of Takotsubo syndrome, at least 2.5% had definitive angiographic evidence of SCAD on expert review [2].

Cardiac CT angiography (CTA) and cardiac MRI can provide complementary diagnostic information, particularly when the diagnosis remains uncertain after echocardiography. Cardiac MRI is valuable in the workup of SIC, as it can demonstrate the classic pattern of myocardial edema on T2-weighted imaging with a notable absence of late gadolinium enhancement (LGE), a feature that helps distinguish SIC from myocarditis and infarction, both of which typically show LGE in a non-ischemic or ischemic distribution, respectively. CTA can also be used to assess for SCAD, particularly for surveillance imaging or in select patients as an alternative to repeat invasive angiography; however, its spatial resolution is lower than that of invasive coronary angiography, and it may miss subtle dissections, particularly those involving distal or smaller-caliber vessels. As such, CTA is generally reserved for non-emergent follow-up evaluation of arterial healing rather than initial diagnosis, and invasive coronary angiography remains the gold standard for confirming SCAD [4].

The management of concurrent SCAD and SIC presents a unique challenge, as the two conditions carry distinct therapeutic considerations. In hemodynamically stable patients with SCAD, a conservative approach is generally preferred, since spontaneous healing occurs in most cases and percutaneous coronary intervention carries an increased risk of dissection propagation and procedural failure [1-3,5]. Beta-blockers are commonly recommended to reduce coronary arterial shear stress and potentially decrease the risk of dissection progression or recurrence [2]. In SIC, angiotensin-converting enzyme inhibitors have been associated with improved outcomes, whereas the benefits of beta-blockers and antiplatelet therapy remain less well established [2].

The prognosis of concurrent SCAD and SIC is generally favorable. Spontaneous healing of the coronary dissection occurs in the majority of cases, and the wall motion abnormalities characteristic of SIC are typically transient with recovery of left ventricular function in most patients. Patients with SCAD experience lower rates of atrial fibrillation, congestive heart failure, and in-hospital mortality compared with patients with isolated TTS [4]. Recurrent SCAD is a known risk, and a follow-up evaluation for underlying arterial abnormalities, such as fibromuscular dysplasia, is recommended in affected patients [2-4]. 

## Conclusions

This case demonstrates the rare and possible coexistence of SCAD and SIC in an older woman presenting with acute coronary syndrome. Although these conditions are distinct, their overlapping clinical, electrocardiographic, and imaging features may complicate diagnosis. Clinicians should therefore consider SCAD in patients with suspected Takotsubo syndrome and carefully evaluate for coronary pathology, even in older patients with conventional cardiovascular risk factors. Their association may reflect a shared catecholamine-mediated response, a common precipitating stressor, or a bidirectional relationship in which one condition triggers the other.

Although SCAD and SIC generally have favorable outcomes with conservative management, this patient developed complete heart block followed by asystolic cardiac arrest. This course underscores the potential for severe electrical instability and the need for close monitoring during the acute phase. Cardiac arrest precluded cardiac magnetic resonance imaging, which might have helped distinguish Takotsubo syndrome from a left anterior descending artery infarction caused by SCAD. Further investigation is needed to clarify the pathophysiologic relationship between these conditions, identify patients at greatest risk for malignant complications, and guide optimal diagnostic and therapeutic strategies.
